# Primary hepatic angiomyolipoma: immunohistochemistry and electron microscopic observations: a case report

**DOI:** 10.1186/s13256-017-1235-1

**Published:** 2017-03-22

**Authors:** Hidefumi Kubo, Hitoshi Yamazaki, Takemichi Okada, Yoshihito Takahashi, Yatsushi Nishi, Hiroaki Yokomori

**Affiliations:** 1 Department of Surgery, Kitasato University Medical Center, Kitamoto, Saitama Japan; 2 Department of Pathology, Kitasato University Medical Center, Kitamoto-Saitama, Japan; 3 Department of Radiology, Kitasato University Medical Center, Kitamoto, Saitama Japan; 4 Department of Internal Medicine, Kitasato University Medical Center, Saitama, Japan

**Keywords:** Hepatic angiomyolipoma, Ethoxybenzyl MRI, Electron microscopy, Immunohistochemistry

## Abstract

**Background:**

Hepatic angiomyolipomas are a rare, benign group of mesenchymal tumors in the liver. Hepatic angiomyolipoma is sometimes misdiagnosed as hepatocellular carcinoma, and there is the possibility of a malignant transformation. Hence, the accurate diagnosis of this disorder is necessary.

**Case presentation:**

A 64-year-old Japanese man was observed to have a space-occupying lesion of 15-mm diameter in the liver during a follow-up examination for a previously resected cecal carcinoma. He underwent lateral segmentectomy of the liver with a provisional diagnosis of hepatic metastatic recurrence of the carcinoma based on gadolinium ethoxybenzyl diethylenetriamine pentaacetic acid-enhanced magnetic resonance imaging and diffusion-weighted imaging.

**Conclusions:**

We have explored the combination of gadolinium ethoxybenzyl diethylenetriamine pentaacetic acid-enhanced magnetic resonance imaging and histological examination to confirm our diagnosis of hepatic angiomyolipoma comprising an intimate mixture of numerous abnormal blood vessels, adipocytes, and epithelioid spindle cells of various sizes. Immunohistochemical examination revealed characteristic pathological findings associated with positive qualitative immunoreactions for human melanoma black 45 and desmin. Electron microscopic findings revealed the presence of melanosomes in the epithelioid cells. Diffusion-weighted imaging provides a more accurate diagnostic image with the characteristic macroscopic appearance of hepatic angiomyolipoma. Through immunohistochemistry and electron microscopy, we also show that this benign tumor comprises tissue elements.

## Background

Angiomyolipoma (AML) is a rare, benign mesenchymal neoplasm most often arising in the kidney. Hepatic angiomyolipoma (HAML) is a rare, benign mesenchymal liver tumor first described by Ishak in 1976 [1]; it belongs to a group of perivascular epithelioid cell (PEC) tumors called *PEComas* [2]. HAML may be derived from PECs [3], which are cells with multiple differentiation potentials that are capable of differentiating into vascular smooth muscle and epithelial cells and expressing the melanoma cell marker human melanoma black (HMB)-45 [4].

Recently, HAML lesions identified by focusing on gadolinium ethoxybenzyl diethylenetriamine pentaacetic acid-enhanced magnetic resonance imaging (Gd-EOB-DTPA MRI), immunohistochemistry, and both clinical and pathological characteristics of the tumors were reported [5]. However, to the best of our knowledge, there is no case report to date on the combination of Gd-EOB-DTPA MRI, immunohistochemistry, and electron microscopy. In this report, we aim to further characterize HAML, in particular that of the epithelioid, the phenotypic smooth muscle spindle type, and adipocytic cells.

## Case presentation

**Fig. 1:**
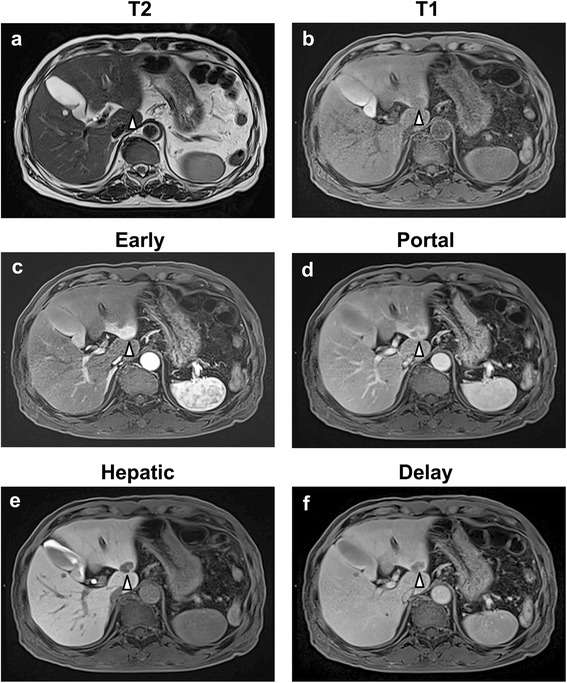
Magnetic resonance imaging (MRI) findings. **a** T2-weighted image with fat saturation showing a heterogeneous hyperintense mass in segment 2 of the right hepatic lobe. **b** T1-weighted image showing the lesion with a homogeneous low signal intensity. **c**–**f** Gadolinium ethoxybenzyl diethylenetriamine pentaacetic acid-enhanced magnetic resonance imaging findings. **c** Marked heterogeneous high signal intensity in the arterial phase of the dynamic gadolinium ethoxybenzyl diethylenetriamine pentaacetic acid-enhanced magnetic resonance imaging scan. **d** The signal intensity is relatively reduced in the portal venous phase, but slightly higher than in the surrounding liver parenchyma, with an enhanced vascular signal visible in the lesion. **e** The signal intensity of the tumor is lower at the parenchymal phase than in the surrounding liver parenchyma. **f** A lack of gadolinium ethoxybenzyl diethylenetriamine pentaacetic acid-enhanced magnetic resonance imaging uptake is noted in the hepatobiliary phase at 40 minutes postinjection. *Arrowheads* denote tumor lesion in segment 2 of liver

A 64-year-old Japanese man with no evidence of tuberous sclerosis was discovered to have a space-occupying lesion in the liver during a follow-up examination for cecal cancer. After ileocecal cancer surgery, no signs of metastasis were detected on imaging examinations, and tumor markers were not detected. No infectious diseases, such as schistosomiasis, were detected, and the patient did not use oral hormonal agents. Physical examination of the patient’s abdomen revealed normal active bowel sounds, no tenderness or rebound tenderness of the epigastrium, and no hepatomegaly or splenomegaly. Results of tests for antibodies against hepatitis B surface antigen and hepatitis C virus were negative. Tumor marker levels, such as α-fetoprotein, protein induced by vitamin K absence or antagonist II, carcinoembryonic antigen, and carbohydrate antigen 19-9, were within the normal ranges. Abdominal ultrasonography revealed a 25-mm low-density tumor with a high-density area in segment 2 (S2) of the liver. Gd-EOB-DTPA MRI also showed a slight T2 hyperintensity (Fig. 1a) and T1 hypointensity 22 × 12-mm tumor (Fig. 1b) in S2 of the liver. Heterogeneous high signal intensity was markedly seen in the arterial phase of the dynamic Gd-EOB-DTPA MRI (Fig. 1c), with multiple central filiform vessels and capsule enhancement. The signal intensity was relatively reduced in the portal venous phase (PVP) (Fig. 1d), but it was slightly higher than that in the surrounding liver parenchyma with an enhanced vascular signal visible in the lesion (Fig. 1e). The signal intensity of the tumor was lower at the parenchymal phase than at the surrounding liver parenchyma (Fig. 1e). A lack of Gd-EOB-DTPA uptake was noted in the hepatobiliary phase (HBP) at 20 minutes postinjection (Fig. 1f).

**Fig. 2:**
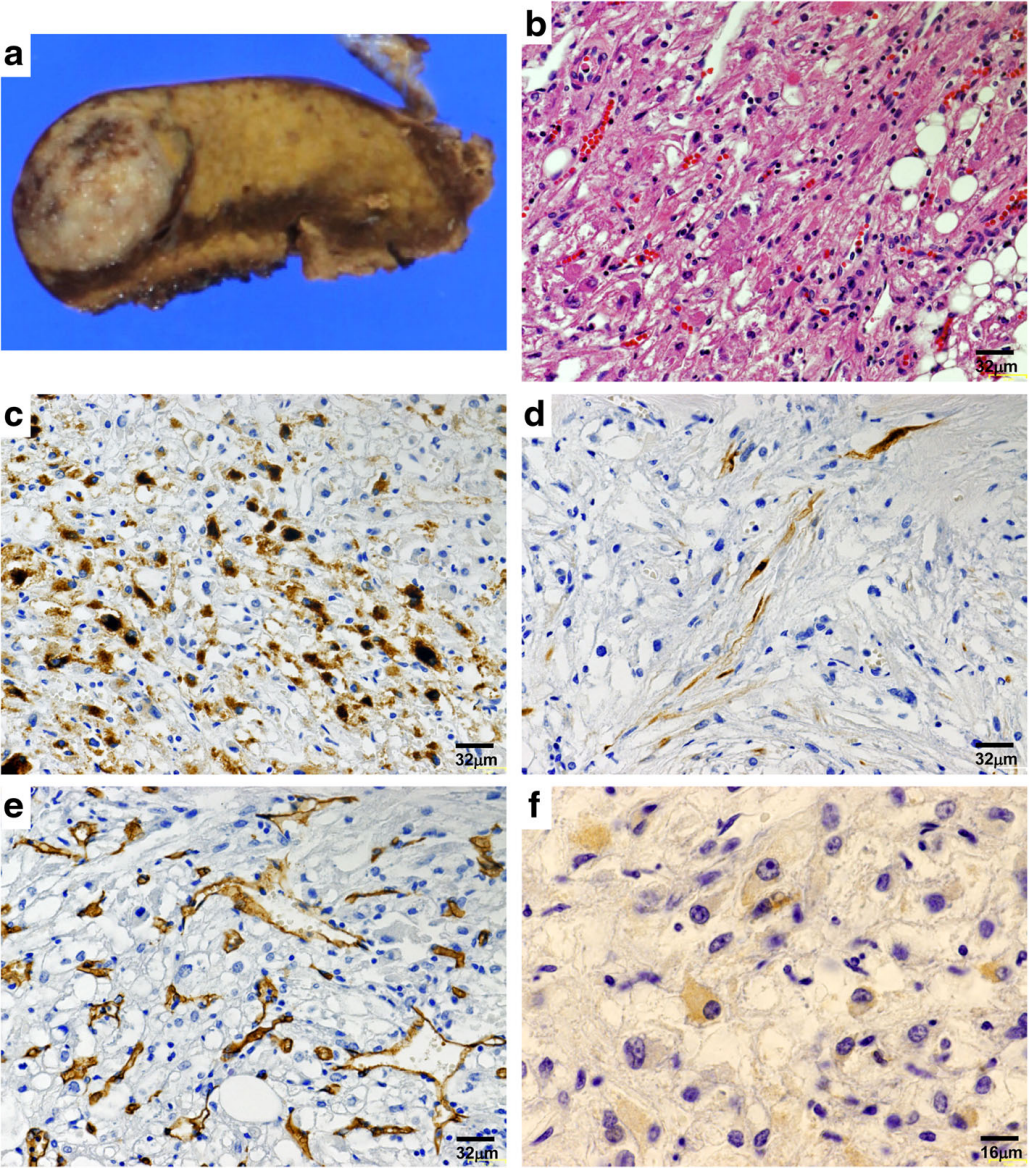
Pathological findings. **a** Macroscopic findings. The excised section of the tumor shows only a solid component, without a cystic component or apparent capsule. **b** Microscopic findings. A tumor without fibrous capsule with interlacing bands of uniform spindle cells, of which elongated nuclei are arranged in a palisading pattern. **c**, **d** Immunohistochemical findings The epithelioid spindle cells are strongly positively stained for (**c**) human melanoma black 45 and (**d**) desmin. **e** CD34 underlined the rich demonstrate abundant mature vascular structures. **f** The tumor cells are focally positive for S100

**Fig. 3:**
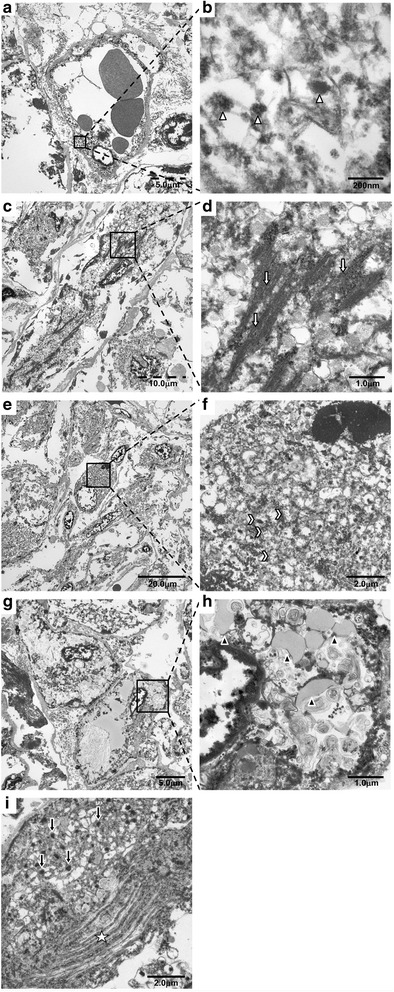
Electron microscopic findings. **a**, **b** Capillary endothelial cells show abnormal thickening of the subendothelial spaces containing collagen fibrils in a homogeneous matrix, fibroblast-like cells, and nonspecific vesicular structures. *White arrowheads* indicate Weibel-Palade bodies. **c**, **d** Smooth muscle cells with abundant accumulation of glycogen and myofilaments, as well as a few dense granules. Myofilaments are dispersed at the periphery of the cell along the cytoplasmic membranes. *White arrows* indicate myofilaments. **e**, **f** Epithelioid cells contained concentric membranous endoplasmic reticulum, numerous mitochondria, and cytoplasmic glycogen granules. *Double arrowheads* indicate mitochondria. **g**, **h** Adipocytic cells occupied by homogeneous osmophilic lipid vacuoles that are not membrane-bound. *Black arrowheads* denote lipid vacuoles. **i** Epithelioid cells contain myelin figures. Melanosomes were present in the epithelioid cells. *Black arrows* denote melanosome. *Star* denotes myelin features in **a**, **c**, **e** and **g** (low magnification). **b**, **d**, **f**, **h**, **i** High-magnification views

With a clinical diagnosis of hepatocellular carcinoma (HCC) and hepatic adenoma, a partial hepatectomy of S2 was performed. The resected specimen showed a tumor of 25 mm in diameter. The cut surface of the resected liver showed a relatively firm, whitish-gray nodule, which was well circumscribed from the surrounding hepatic parenchyma. Some very small, yellowish areas were embedded at the periphery. No distinct necrotic areas were noted (Fig. 2a). A hematoxylin and eosin-stained section showed growth of eosinophilic ovoid cells and spindle cells with focal admixture of mature adipocytes (Fig. 2b). A number of vascular channels were intermingled. Irregular dilation and thickening of the vascular wall were occasionally observed. Immunohistochemical analysis revealed eosinophilic ovoid cells positive for HMB-45 (Fig. 2c) and Melan-A. In contrast, spindle cells were positive for desmin (Fig. 2d). Vigorous staining of CD34 exhibited a dense vascular network of the tumor (Fig. 2e). Moreover, the tumor cells were focally positive for S100 (Fig. 2f). In some cases, useful information has been obtained by transmission electron microscopy after tissue collection. We investigated the reembedding of formalin-fixed and paraffin-embedded specimens with epon resin. HAMLs, each composed of an intimate mixture of numerous abnormal blood vessels with fat, spindle, and/or epithelioid cells of various sizes, were found on electron microscopic examination. Capillary endothelial cells with Weibel-Palade bodies showed abnormal thickening of the subendothelial spaces containing collagen fibrils in a homogeneous matrix, fibroblast-like cells, and nonspecific vesicular structures. These findings suggest secondary vascular damage; the thickened vessels may not be a primary, integral part of HAML (Fig. 3a and b). Smooth muscle cells showed abundant accumulation of glycogen and myofilaments with a few dense granules (Fig. 3c and d). Myofilaments were typically dispersed at the periphery of the cell along the cytoplasmic membranes (Fig. 3c and d). Some epithelioid cells contained concentric membranous endoplasmic reticulum, numerous mitochondria, and cytoplasmic glycogen granules (Fig. 3e and f). In adipocytic cells, the cytoplasm was occupied by homogeneous osmophilic lipid vacuoles that were not membrane-bound. Occasional cells were multivacuolar (Fig. 3g and h). Cytoplasmic myelin figures and melanosomes were present in epithelioid cells (Fig. 3i). We diagnosed type I mixed type HAML. The patient was alive with no tumor recurrence or metastasis at the 1-year follow-up.

## Discussion

Most of the patients with HAML have no symptoms and their lesions are detected incidentally during routine medical examination [6]. The typical appearance of HAML is that of a smoothly contoured, heterogeneous, high-echo lesion. Our patient had a history of cecal cancer surgery, and HAML was detected incidentally during a routine follow-up imaging examination. Gd-EOB-MRI also confirmed a large tumor in the caudate lobe and numerous smaller nodules in the right lobe of the liver. A combination of Gd-EOB-MRI and diffusion-weighted imaging (DWI) provided even higher diagnostic sensitivity. Last, the characteristic macroscopic appearance and histopathology provided a definitive diagnosis.

HAML comprises three tissue elements: blood vessels, epithelia or spindle-shaped smooth muscle cells, and mature adipose tissue. Immunohistochemical staining showed absence of hepatocytes, but smooth muscle cells were strongly positive for a melanocytic cell-specific monoclonal antibody. The final histopathological diagnosis (HMB-45-positive) is consistent with HAML. Moreover, formalin-fixed tissue sections examined under an electron microscope showed moderate amounts of stacked, rough endoplasmic reticulum; mitochondria; lysosomes; and oval, cytoplasmic, melanosome-like, electron-dense granules.

According to the differential diagnosis, we first investigated HAML using Gd-EOB-DTPA MRI, immunohistochemical analyses, and electron microscopy. Type I hybrid-type lesions were enhanced in the arterial phase but were followed by rapid washout in the PVP and delayed phases.

Because the radiological features of HAML depend on the relative proportion of adipose cells in the tumor, HAML was not readily distinguishable from HCC [5–8]. Liver biopsy was helpful in the preoperative diagnosis of AML [9]. In this case, it was difficult to perform liver biopsy because the tumor was located just below the liver capsule. In the present case, we suspected HCC and hepatocellular adenoma.

The imaging characteristics of HAML correlated with its histological components, and detection of fat content was typical of HAML. MRI is more sensitive than computed tomography in detecting fatty tissue, which appears hypointense on fat saturation pulses or chemical shift imaging. However, it is occasionally difficult to differentiate between HAML and other hepatic tumors that contain fatty tissue, such as fatty metamorphosis of HCC, hepatocellular adenoma with fat content, lipoma, and liposarcoma [8, 9].

The MRI findings reported by Sakamoto were of a low-signal-intensity mass with some tiny, high-signal-intensity foci on T1-weighted images. The tiny, high-signal-intensity foci were thought to be due to abundant adipose tissues, as indicated by the results of immunohistochemical examination [10].

Moreover, according to a recent study in which researchers compared Gd-EOB-MRI features of lipid-poor HAMLs and HCCs, both lesions showed similar dynamic enhancement patterns during the arterial phase, followed by hypointensity on PVP or transitional phase (late dynamic phase) [11]. However, considering that HAMLs are devoid of hepatocytes, whereas HCCs do contain hepatocytes with various degrees of malignant change, homogeneous hypointensity of the mass on HBP imaging and a lower value of relative signal intensity on HBP compared with that of the spleen may suggest hepatic AML rather than HCC [11]. HAML can be divided into four groups based on the tissue components (thick-walled vessels, adipose tissue epithelioid smooth muscle cell, and four subtypes: [1] hybrid: the most common type, which contains similar proportions of each tissue element within the tumor; [2] myoma: smooth muscle tissue, which is the dominant element; [3] lipoma: adipose tissue, which is the dominant element; and [4] hemangioma: dominated by the vascular element) [12]. In our patient, T2-weighted MRI sequences showed slight hyperintensity, whereas T1-weighted MRI sequences showed a hypointense tumor. Heterogeneous high signal intensity was particularly observed in the arterial phase of the dynamic Gd-EOB-DTPA MRI sequence with multiple central filiform vessels and capsular enhancement. The signal intensity was relatively reduced in the PVP. A lack of Gd-EOB-DTPA uptake was noted in the HBP at 20 minutes postinjection; in our patient, the tumor was thought to be of the hybrid mixed type.

Considering the developmental site of HAML, in our patient, because immunohistochemical analyses and electron microscopy revealed all three components of an AML (vascular cells, immature smooth muscle cells, and fat cells) and because three differentiated epithelioid cells contained a so-called secondary hit mutation, they were believed to have derived from a common progenitor cell [13]. Earlier reports described ultrastructural findings of HAML cells [14, 15], but no report of the relevant literature has explained the structure of myelin figures. In our patient, electron microscopy revealed the presence of cytoplasmic myelin figures and melanosomes in the epithelioid cells. These cells were derived from undifferentiated cells of neural cast [13]. Generally, S100 staining was negative in HAML. Nevertheless, some cases of immunoexpression of S100 by HAML have been reported [16]. Although they were not immunohistochemically detected on the neural cell, electron microscopy revealed myelin figures in the cytoplasm or as inclusions in mitochondria and autophagic vacuoles, where they might represent artifacts of lipid fixation [17]. Two hypotheses have been proposed to explain the histogenesis of HAML. One is that HAMLs derive from undifferentiated cells of the neural crest that can express dual smooth muscle and melanocytic phenotypes. The second is that these tumors are of myoblastic and, more specifically, smooth muscle origin, but that they are molecularly altered. They coexpress melanogenesis and melanocytic markers [13]. Recently, telocytes have been shown to give rise, in several organs, to a spectrum of lesions that variably express at least some of the cell differentiation markers [18].

HAML is generally managed as a benign tumor in clinical settings; however, since 2000, several reports have revealed its potential for malignant transformation with evidence of recurrence [19, 20]. Maklouf *et al*. showed that CD117 is positive in all cases of benign renal AML and HAML [21]. In our patient, the tissue stained positive for CD117 (data not shown). Therefore, a consensus on the optimal treatment approach for HAML is yet to be established. Actually, HAML comprises a rare, benign group of mesenchymal tumors in the liver. Moreover, its cause remains unclear. We also elucidated five electron microscopic features of HAML in our patient. Vascular and smooth muscle components were monoclonal, whereas three types of differentiated epithelioid cells of fat tissue components were polyclonal. This finding suggests that the tumor cells derived from HAMLs were a type of primitive mesenchymal cell with multidifferentiation potential. These cells might differentiate into vascular smooth muscle cells and adipocytes [13].

According to the future plan, no signs of tumor recurrence or metastasis were observed during the follow-up period. Therefore, HAML should not be considered as an entirely benign tumor; at least, there is a possibility of its malignant transformation. We will continue with periodic imaging examinations for our patient.

## Conclusions

We have explored the combination of Gd-EOB-DTPA MRI and DWI, which provides a more accurate diagnostic picture with the characteristic macroscopic appearance of HAML. Moreover, HAML is sometimes misdiagnosed as HCC. This may lead to its malignant transformation. Hence, its accurate diagnosis is necessary. Through immunohistochemistry and electron microscopy, we have also shown that this benign tumor comprises tissue elements.

## Abbreviations

AML: Angiomyolipoma
DWI: Diffusion-weighted imaging
Gd-EOB-DTPA MRI: Gadolinium ethoxybenzyl diethylenetriamine pentaacetic acid-enhanced magnetic resonance imaging
HAML: Hepatic angiomyolipoma
HBP: Hepatobiliary phase
HCC: Hepatocellular carcinoma
HMB-45: Human melanoma black 45
PEC: Perivascular epithelioid cell
PVP: Portal venous phase
